# Resilience and evolutionary insights in PPI networks: comparative analysis of node resilience and centrality measures

**DOI:** 10.3389/fgene.2025.1613475

**Published:** 2026-01-07

**Authors:** Jiarui Zhang

**Affiliations:** School of Artificial Intelligence and Information Engineering, Yichun University, Yichun, China

**Keywords:** biological networks, network structure, network resilience, node resilience, centrality measures

## Abstract

**Introduction:**

Protein-protein interaction (PPI) networks serve as the central framework for deciphering the modular structure of cellular functions and signal transduction mechanisms. While established network topological Measures (such as degree centrality, betweenness centrality, and closeness centrality) can statically characterize nodal connectivity density or pathway intermediation capacity, they fail to dynamically capture cascade following node failure.

**Method:**

This study employs systems biology approaches to quantitatively analyze network resilience based on bacterial PPI network data obtained from the Stanford Network Analysis Platform (SNAP). First, a progressive node removal strategy was implemented to simulate cascading failure propagation and evaluate system-level resilience degradation dynamics. Subsequently, single-node knockout experiments were systematically conducted to quantify local topological disruption effects, with network fragmentation metrics (e.g., giant component size decay rate) being integrated to establish the Node Resilience (NR) index. To validate the biological relevance of NR, we developed a multidimensional analytical framework that performs cross-correlation analysis between NR and classical centrality measures [Degree centrality (DC), Betweenness centrality (BC), Closeness centrality (CC), Eigenvector centrality (EC)], enabling systematic revelation of consensus vital nodes identified by both approaches, and unique sensitive nodes detectable only through resilience-oriented perturbation analysis.

**Results and Discussion:**

Our systematic node removal simulations revealed biphasic resilience degradation across bacterial PPI networks: progressive node failure induced gradual resilience decay whereas exceeding a critical threshold for each network triggered accelerated collapse. This phase transition aligns with evolutionary design principles - modular architectures buffer localized perturbations through functional redundancy, but inter-modular bridge depletion beyond criticality propagates cascading failures via weakly coupled connections. Notably, NR exhibited a strong negative correlation with BC, contrasting with weak associations for DC, CC, and EC. This dichotomy arises because BC quantifies cross-modular information brokerage - high-BC nodes act as structural keystones whose removal disconnects functional modules, drastically reducing global entropy. Conversely, for DC, CC, and EC primarily reflect local connectivity patterns with limited cascade propagation potential.

## 1 Introduction

Biological networks, as abstract representations of molecular interactions in living systems, encompass diverse subtypes including metabolic pathways, gene regulatory networks, and protein-protein interaction (PPI) networks. Research on general biological networks has laid the groundwork for understanding molecular interaction systems. As early as the 1990s, the analysis of metabolic pathways originated in the 1990s and established essential methods for network structure and function analysis (Fell and Cornish-Bowden, 1997). Subsequently, Barabási and Oltvai formalized the network biology theory, revealing that biological networks (including PPI networks) exhibit scale-free, hierarchical modular, and ultra-small-world topological principles that underpin their functional resilience and evolutionary dynamics (Barabasi and Oltvai, 2004).

Protein-protein interaction (PPI) networks serve as foundational frameworks in systems biology, encoding the intricate functional relationships that sustain cellular processes (Ciliberti et al., 2007). PPI networks enable systematic interrogation of protein complexes, signaling cascades, and functional modules, elucidating regulatory architectures governing cellular processes (Gavin et al., 2002; Von Mering et al., 2002; Ideker and Krogan, 2012). Their analysis has thus become indispensable for deciphering biological complexity in proteomics and systems biology (Wodak et al., 2013; Szklarczyk et al., 2019).

External perturbations, sucn as genetic mutations, pathogenic invasions, or pharmacological agents, can destabilize PPI network topology and functionality (Albert et al., 2000; Rodrigues et al., 2021). Node removal (e.g., protein dysfunction) disrupts associated edges, isolating the node from its interactome. Progressive node loss exacerbates topological disintegration, compromising critical functional modules and system-wide stability (Jeong et al., 2000; Motter, 2004). Quantifying these dynamics is pivotal for understanding disease pathogenesis, therapeutic efficacy, and evolutionary adaptation (Goh et al., 2007).

Zitnik et al. (2019) conducted evolutionary analyses of 1,840 species and found that phylogenetically advanced organisms exhibit enhanced PPI network resilience, suggesting that natural selection favors robust topologies to buffer environmental stresses. However, the precise mechanisms governing resilience decay under sustained intensifying perturbations, particularly in microbial systems, remain poorly understood. Addressing this, we present a computational framework to model progressive node removal in bacterial PPI networks, integrating entropy-based resilience measures and iterative simulations to dissect fragility dynamics. Our study identifies biphasic resilience degradation across bacterial interactomes: gradual decay under low-intensity perturbations transitions abruptly to systemic collapse when node failure surpasses a critical threshold. This phase transition, characterized by steepened decay slopes, underscores the evolutionary trade-off between metabolic efficiency and redundancy allocation.

Furthermore, we introduce Node Resilience (NR), a novel metric quantifying individual proteins’ contributions to global network stability. Comparative analyses with traditional centrality measures reveal that NR inversely correlates with betweenness centrality (Joyce et al., 2010), highlighting bridge nodes as structural keystones whose removal triggers modular disarticulation.

To illustrate the significance of resilience-critical nodes, we conducted a case study on *Listeria monocytogenes*, demonstrating that these nodes frequently orchestrate virulence regulation and metabolic adaptation. These findings advance our understanding of PPI network evolvability, offering insights into therapeutic targeting and the evolutionary drivers of interactome robustness.

## 2 Methods

### 2.1 Network resilience

PPI networks represent functional associations between proteins within a biological system (Barabasi and Oltvai, 2004). Nodes denote individual proteins, while edges signify pairwise interactions. These networks are typically undirected and unweighted. Notably, the PPI networks in this study are derived from the largest connected component (LCC). Zitnik et al. formalized resilience as the network’s ability to sustain connectivity under iterative perturbations in 2019.

The network is progressively fragmented by iteratively isolating nodes, that is, removing all their links. Links represent protein-protein interactions, and their removal result in isolated proteins and smaller non-interacting components. The improved Shannon diversity index is employed to quantify the diversity of components in a network after nodes removal. The process involves removing nodes from the originally connected network G , which subsequently fragments into k disconnected components. The index is calculated as Equation 1:

$$H_{msh}\left(G_{f}\right)=-\frac{1}{\log N}\sum_{i=1}^{k}p_{i}\log\;p_{i}$$ (1)

where *N* is the total number of nodes in the original network *G*, *k* is the total number of components in the fragmented network *G*_*f*_ *, p*_*i*_ *= |c*_*i*_ *|/N* is the proportion of nodes in component *c*_*i*_ relative to *N*. Therefore, *p*_*i*_ represents the probability that a randomly selected node belongs to the connected component *c*_*i*_.

The modified Shannon diversity *H*_*msh*_ was employed to quantify the component diversity of the protein interactome under increasing node failure levels *f*. At *f* = 0, the network is intact and *H*_*msh*_ = 0. As f increases and more proteins are removed, the network disintegrates and *H*_*msh*_ rises. When all proteins are isolated (*f* = 1), *H*_*msh*_ peaks at 1.

The resilience metric integrates entropy across perturbation intensities. This integration quantifies the network’s ability to maintain stability under different disturbance levels, and its calculation is defined by Equation 2:

$$R\left(G\right)=1-\int_{0}^{1}H_{\text{msh}}\left(G_{f}\right)d\;f$$ (2)

where *H*_*msh*_ *(G*_*f*_ *)* denotes entropy at failure rate *f*. A high *R(G)* value reflects delayed fragmentation during progressive node removal, indicating networks that retain a giant connected component despite cumulative perturbations.

Broad-scale evolutionary studies across diverse taxa have demonstrated that organisms positioned higher on the phylogenetic scale develop reinforced interactome robustness (Zitnik et al., 2019). Experimental validation from microbial evolution studies, including the *Escherichia coli* Long-Term Evolution Experiment, demonstrates that evolved strains exhibit PPI networks with significantly higher resilience than randomized counterparts, demonstrating selective maintenance of network stability under prolonged environmental pressures (Maddamsetti, 2021). These findings posit resilience as both a product of functional constraints and an evolutionary driver, enabling survival in fluctuating ecological niches.

### 2.2 Node resilience

In PPI networks, spatially heterogeneous nodes exhibit marked functional heterogeneity, while the systematic identification of topological hubs under dynamic environmental perturbations, remains a central unresolved challenge. Although traditional centrality measures [e.g., degree centrality, closeness centrality (Freeman, 1978), eigenvector centrality (Bonacich, 1987), and betweenness centrality (Freeman, 1977)] can effectively predict essential proteins, they exhibit systematic limitations in quantifying the cascade disruption effects induced by node removal on network connectivity.

To quantify the contribution of individual nodes to network resilience, we introduce the concept of Node Resilience (*NR*). This metric evaluates the criticality of a node by measuring the change in network resilience after its removal. The calculation process is as follows: Consider a PPI network *G*, represented as *G=(V,E)*, where *V* is the set of *N* nodes (*v*_*i*_ *∈V, i = 1,2,…,N)*, and *E* is the set of edges representing interactions between nodes.

For each node *v*_*i*_ in *G*, its removal results in a new network *G'=(V|{v*_*i*_ *},E′)*, where *E′* is the set of edges after removing all interactions involving *v*_*i*_. The resilience of the residual network *G′* is then calculated, yielding the resilience value for node *v*_*i*_, denoted as *NR*_*i*_.

The complete set of node resilience values {*NR*_*1*_ *,NR*_*2*_ *,…,NR*_*n*_ } is generated by iteratively removing each node *v*_*i*_ from original network *G* and quantifying the resilience *R* of the resulting residual network *G|{v*_*i*_ *}*, formally expressed as Equation 3:

$$\left\{NR_{1},...,NR_{n}\right\}=\left\{R\left(G|\left\{v_{i}\right\}\right)\left|\forall \right.v_{i}\in V\right\}$$ (3)

## 3 Results

### 3.1 Progressive degradation of network resilience under node removal

Systematic interrogation of how PPI networks degrade under escalating perturbations (a critical determinant of cellular robustness) remains limited. Here, we dissect resilience dynamics in bacterial interactomes through iterative node failure simulations, probing both evolutionary design rules and catastrophic fragility thresholds.

We developed a computational framework to model progressive network degradation via stochastic node removal, wherein node removal rates (f) increased incrementally from 0% to 100% in 1% steps, simulating evolutionary stressors such as pathogenic gene silencing. Network resilience was quantified using the entropy-integrated, which captures a network’s capacity to buffer perturbations. To ensure statistical reliability, each failure rate underwent 100 independent iterative simulations.

**FIGURE 1:**
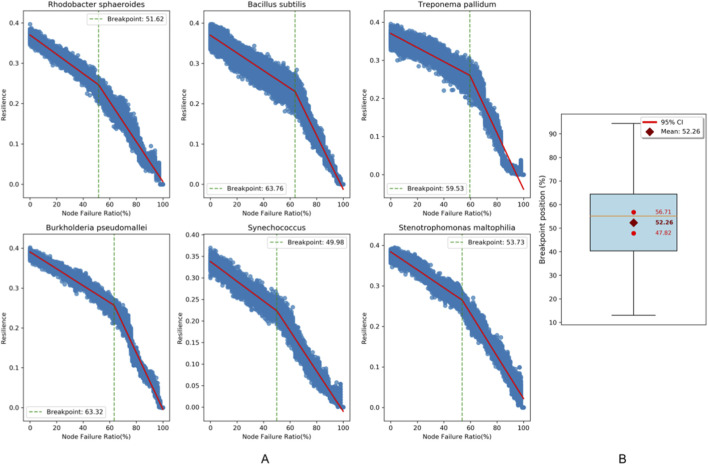
Resilience degradation dynamics in bacterial PPI networks under progressive node failure rate. **(A)** The six scatter plots on the left illustrate the resilience variation trends of six distinct bacterial species during the progressive node removal process (with 1% removal per step). In each plot, the red fitted line intuitively depicts the change pattern of resilience with the Node Failure Ratio. A breakpoint, marked by a green dashed line, exists in all plots. **(B)** The box plot on the right summarizes the distribution range of resilience breakpoints across all bacteria. The results show that the breakpoints are concentrated in the Node Failure Ratio interval of 40%–65%.

We analyzed six bacterial protein-protein interaction (PPI) networks as described in Table 1, which were retrieved from the SNAP database (Figure 1A). The six bacteria cover multiple key evolutionary branches of the bacterial domain, with distinct biological functions and survival strategies, enabling comprehensive verification of network resilience trend across different functional bacterial types.

**TABLE 1:** Network and computational characteristics of bacterial PPI networks.

NCBI taxon ID	Bacteria	Nodes	Edges	Runtime (network resilience degradation) (s)	Breakpoints (%)	Runtime (node resilience analysis) (s)	Outlier nodes
349102	Rhodobacter sphaeroides	684	3093	33	51.62	573	5.3
224308	Bacillus subtilis	1293	5507	61	63.76	1911	5.1
243276	Treponema pallidum	641	1613	22	59.53	322	5.0
272560	Burkholderia pseudomallei	1178	7304	69	63.32	2028	5.1
321332	Synechococcus	466	1543	18	49.98	208	5.2
391008	Stenotrophomonas maltophilia	655	2992	34	53.73	520	5.2

**FIGURE 2:**
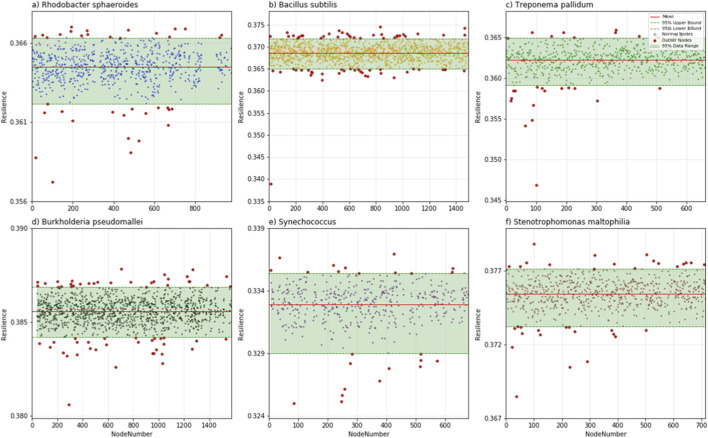
Node resilience of the six bacterial PPI networks. In each subgraph, the horizontal axis represents the order of nodes’ appearance in the PPI network, while the vertical axis denotes node resilience (quantifying the network’s resilience after the removal of individual nodes). As shown in the figure, most nodes have resilience values falling within the 95% confidence interval, with only a small fraction lying outside this range-these outlier nodes are highlighted in red. This indicates that the resilience values of most nodes show little variation, while the red-highlighted subset exhibits significant deviations.

All computational time data presented in this table were collected on an ecs. g9i.xlarge cloud server instance, which is configured with 4 vCPUs and 16 GiB of memory. Breakpoints denote the resilience threshold of significant network resilience degradation in Figure 1. Outlier Nodes refer to the discrete points with node resilience values far from the central region in Figure 2.

The resilience trajectories of six representative bacterial PPI networks are visualized through progressive node removal simulations. The results reveal the network resilience decreases relatively gently to the left of the breakpoint, while the decline rate accelerates significantly to the right, reflecting the “resilience threshold” characteristic of the network structure. Expanded analysis of bacterial PPI networks with node counts exceeding 100 from the SNAP Database demonstrated measurable criticality in resilience dynamics (Figure 1B). The metabolic networks of these bacteria can maintain resilience through structural redundancy before approximately 40%–65% of nodes are removed, and then lose stability rapidly beyond this threshold.

This study reveals that PPI networks exhibit biphasic dynamics: they buffer low-intensity perturbations, until damage exceeds a critical threshold, where cascading failures drive abrupt robustness collapse (Cohen et al., 2000; Motter, 2004). This phenomenon reflects the robustness-efficiency trade-off strategy evolved in biological systems. The modular architecture achieves effective buffering against stochastic noise under metabolic cost constraints through spatial compartmentalization that restricts local disturbance propagation and redundant functional backups (sparing critical components). When incurred damage surpasses the critical threshold, weakly coupled inter-modular connections may transform into propagation channels for cascade failures, ultimately triggering systemic collapse (Callaway et al., 2000; Gao et al., 2016).

### 3.2 Node resilience versus centrality metrics

We calculated the six bacterial species mentioned above and found that most nodes in the network have a limited impact on overall resilience when removed. However, the removal of a small subset of key nodes, causes significant declines in network resilience and severe fragmentation of the network structure (Figure 2). From Figure 2, we can distinctly observe that across all six bacterial PPI networks, the node resilience (NR) values exhibit a remarkable pattern: the vast majority of nodes cluster showing only marginal fluctuations. In contrast, a small subset of nodes deviates significantly from this cluster, displaying notably higher or lower resilience values. This distribution pattern implies that the network’s overall resilience is robust to the removal of most nodes—their elimination barely perturbs the network’s ability to maintain structural and functional integrity. Conversely, the removal of those few outlying nodes, which possess exceptionally high or low node resilience. This observation aligns with the modular and hierarchical nature of PPI networks, where most nodes participate in redundant or auxiliary interactions, while a select few mediate critical pathways or structural connections that underpin the network’s resilience.

Comprehensive correlation analyses demonstrated that NR captures distinct aspects of topological vulnerability compared to traditional centrality paradigms. A pronounced negative correlation emerged between NR and BC, indicating that nodes functioning as inter-modular bridges—those with high BC values—exhibit disproportionately low resilience. In contrast, NR displayed substantially weaker correlations with DC, CC, and EC.

**FIGURE 3:**
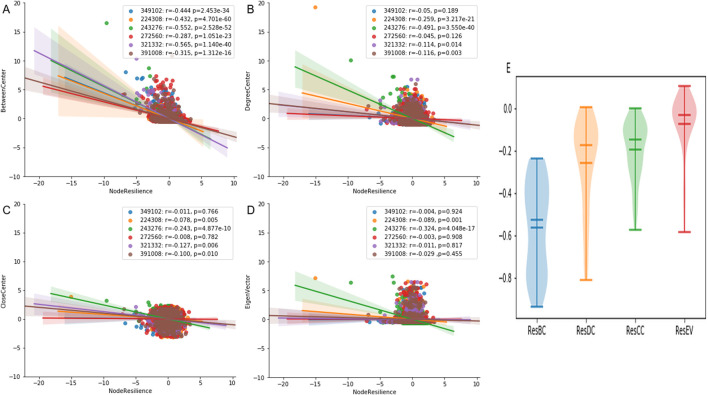
Comparison of NR with Four Centrality measures. **(A–D)** Scatter plots representing the relationship between node resilience and various centrality measures, including DC,CC, BC, and EC. **(E)** Violin plot displaying the distribution of correlation coefficients between node resilience and the centrality measures across all 84 bacterial species from the SNAP database.

To assess the generalizability of these findings, we expanded our analysis to all 84 bacterial species from the SNAP database. The violin plot in Figure 3E summarizes the distribution of correlation coefficients across this extended dataset. BC maintained a robust negative correlation with NR, while DC, CC, and EC showed consistently weak anti-correlations.

The resilience-connectivity duality in PPI networks operates through distinct mechanistic pathways. BC quantifies a node’s role as a bridge in shortest paths—a topological property intrinsically linked to network modular architecture. These bridge nodes facilitate communication between network modules, and their removal induces catastrophic modular disarticulation, effectively fragmenting the network into isolated components. High-BC nodes act as structural keystones whose removal disrupts inter-module communication, effectively decoupling functional units like metabolic pathways or signal transduction cascades (Guimera and Nunes Amaral, 2005; Newman, 2006). This explains their disproportionate impact on network resilience, as measured by NR. Conversely, peripheral nodes with low BC values primarily contribute to intra-modular connectivity. Their removal induces localized rather than whole effects, resulting in minimal NR deviations. While DC, CC, and EC effectively map nodes’ local connectivity influence, proximity to network cores, or leadership within densely connected clusters (Rayfield et al., 2011), they fail to capture the cross-modular integration capacity epitomized by BC. This analytical gap positions NR as a complementary metric that specifically highlights nodes orchestrating global network coherence—those whose removal triggers cascading failure through modular decoupling rather than localized connectivity loss.

### 3.3 A comparative study of NR and BC in the *Listeria monocytogenes*

**FIGURE 4:**
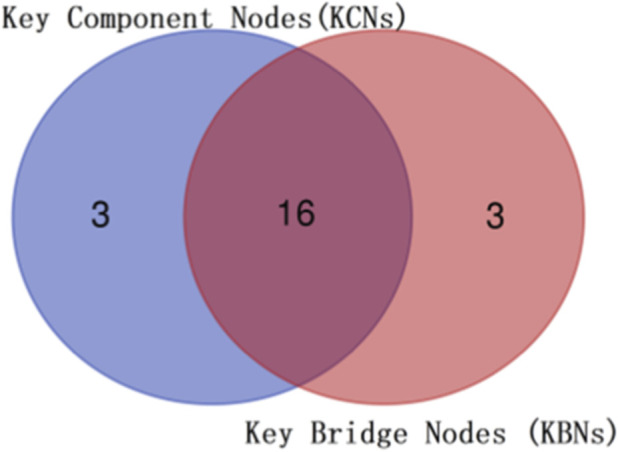
Intersection of KCNs and KBNs datasets in the *Listeria* PPI network: the blue area represents KCNs, and the red area represents KBNs. The overlapping area highlights the dual core nodes.

Our topological analysis of the PPI network of *Listeria monocytogenes* focused on its largest connected compenent (187 nodes, 341 edges) to elucidate core architectural features. Two complementary topological ranking strategies were implemented: NR identified Key Component Nodes (KCNs) — the 10% of nodes whose removal caused maximal resilience loss—while BC pinpointed Key Bridge Nodes (KBNs) — the 10% most frequent mediators of shortest paths. The Venn diagram (Figure 4) illustrates that there is an 89.4% overlap (16 out of 19 nodes) between KCNs and KBNs, highlighting their dual critical roles in structural robustness and information routing. The details of the Key Component Nodes are provided in Table 2.

**TABLE 2:** Key Nodes information.

Gene ID	Gene name	Rank in BC list	Function
lmo1570	pykA	3	Encodes pyruvate kinase and plays an important role in energy supply related to cell division and cell wall synthesis
lmo1053	pdhB	1	Pyruvate dehydrogenase gene
lmo2611	adk	14	Catalyzes the reversible transfer of the terminal phosphate group between ATP and AMP
lmo1348	gcvT	2	The glycine cleavage system catalyzes the degradation of glycine
lmo2367	pgi	15	Catalyzes the reversible isomerization of glucose-6-phosphate to fructose-6-phosphate
lmo2455	eno	7	Catalyzes the reversible conversion of 2-phosphoglycerate (2-PG) into phosphoenolpyruvate
lmo2458	pgk	10	​
lmo1915	lmo1915	9	​
lmo2459	gap	11	Blocks Rab5a-mediated phagosome-endosome fusion
lmo0411	lmo0411	8	​
lmo1437	asd	16	Catalyzes reductive dephosphorylation of L-aspartyl-4-phosphate
lmo1874	thyA	4	Catalyzes dUMP reductive methylation to dTMP using mTHF as methyl donor/reductant
lmo1832	pyrF	17	Catalyzes the decarboxylation of orotidine 5′-monophosphate (OMP) to uridine 5′-monophosphate (UMP)
lmo1929	ndk	5	Major role in the synthesis of nucleoside triphosphates other than ATP
lmo1766	purN	37	​
lmo1831	pyrE	18	Catalyzes the transfer of a ribosyl phosphate group from 5-phosphoribose 1-diphosphate to orotate to form orotidine monophosphate (OMP)
lmo1360	folD	31	Catalyzes the oxidation of 5,10-methylenetetrahydrofolate to 5,10-methenyltetrahydrofolate and its subsequent hydrolysis to 10-formyltetrahydrofolate
lmo1833	pyrD	20	Catalyzes the conversion of dihydroorotate to orotate with NAD + as electron acceptor
lmo2547	hom	19	Involves in amino acid biosynthesis including serine and threonine

The core nodes shared by KCNs and KBNs (e.g., pykA (lmo1570),pdhB (lmo1053), gcvT (lmo1348), thyA (lmo1874), ndk (lmo1929), among others) are extensively involved in critical pathways. Specifically, PykA maintains cell membrane integrity by enhancing fatty acid synthesis (Liu et al., 2017); pdhB conserves resources by downregulating energy metabolism (Stasiewicz et al., 2011); gcvT participates in amino acid metabolic reprogramming (Mujahid et al., 2013); thyA ensures genetic stability by supporting nucleotide synthesis (Kimman et al., 2008); and ndk acts as a pleiotropic hub, simultaneously coordinating nucleotide metabolic balance and virulence expression (Yu et al., 2017). Notably, folD is exclusively present in KCNS and the bifunctional enzyme encoded by the gene is a central component of folate-mediated one-carbon metabolism and directly supports rapid bacterial growth and proliferation (Zhang et al., 2022). Together, these genes form a core network enabling bacterial stress response and enhanced pathogenicity.

## 4 Discussion

This study introduced a new network science approach to evaluate the resilience of PPI networks under simulated disruptions. By constructing a model with randomly removed nodes, we investigated the ability of these networks to maintain topological stability in the face of faults or attacks. Our findings revealed a biphasic resilience degradation pattern: in the pre-critical phase, the network demonstrated an evolutionary adaptation to buffer random failures; however, once node failure reaches the critical threshold, the network exhibits accelerated collapse and inherent vulnerability under continued perturbations. The identification of this critical failure threshold aligns with theoretical models positing that biological networks evolve to maximize robustness against common perturbations while tolerating rare, catastrophic events. This dual-phase behavior underscores the delicate balance between resilience and fragility in biological systems, reflecting their evolutionary optimization for both stability and adaptability.

We proposed a novel metric node resilience (NR), which calculates the resilience value of the network after the removal of each node. This metric quantifies the impact of individual nodes on overall network stability and provides insights into the structural and functional dynamics of PPI networks. The results demonstrate that most nodes in the network have a limited impact on overall resilience when removed. However, the removal of a small subset of key nodes, causes significant declines in network resilience and severe fragmentation of the network structure. These critical nodes play essential roles in connecting different components of the network and maintaining its global connectivity. Their removal disrupts network cohesion, underscoring their importance for preserving biological system functions.

NR is a valuable complement to traditional centrality measures, with distinct correlations tied to network connectivity and modular isolation: a strong negative correlation with BC, as high-BC inter-modular bridges drive systemic fragmentation when removed; a weak negative correlation with DC, since high-DC local hubs only affect intra-modular connectivity and cause minimal isolation; a weak negative correlation with CC, as proximity to cores does not equate to maintaining inter-module links; and a negligible negative correlation with EC, as EC overlooks cross-modular integration—NR’s key focus for resisting modular isolation. While measures like betweenness centrality identify key bridging nodes, node resilience quantifies how the removal of specific nodes affects the entire network’s structural integrity. This dual perspective enriches our understanding of the roles of individual nodes in PPI networks and their contributions to maintaining stability and connectivity.

Despite the promising insights offered by this approach, there are limitations to this study. First, the dataset used is restricted to bacterial organisms, and future research should validate these findings across diverse species and experimental conditions. Second, as network scale increases, computational time grows significantly, which is a scalability limitation of our current framework. Third, for large-scale PPI networks, focusing solely on the impact of individual node failure on network stability has obvious limitations. Our subsequent research will employ protein grouping to systematically categorize nodes, followed by exploring the dynamic changes in network resilience with functional groups as the research unit, which not only addresses the inadequacy of single-node analysis but also provides a more biologically meaningful perspective on how functional modules collectively influence network stability while mitigating the computational burden associated with large-scale networks.

In summary, the use of node resilience as a new metric for assessing the contribution of individual nodes to network stability represents a significant advancement in understanding and analyzing complex biological systems. This approach complements existing network analysis techniques and opens new avenues for studying PPI networks, with potential applications in systems biology, disease modeling, and therapeutic target identification.

## Data Availability

Publicly available datasets were analyzed in this study. This data can be found here: http://snap.stanford.edu/tree-of-life/.
